# Space filling of β-cyclodextrin and β-cyclodextrin derivatives by volatile hydrophobic guests

**DOI:** 10.3762/bjoc.9.133

**Published:** 2013-06-19

**Authors:** Sophie Fourmentin, Anca Ciobanu, David Landy, Gerhard Wenz

**Affiliations:** 1 University Lille Nord de France, F-59000 Lille, France; 2 ULCO, UCEIV, F-59140 Dunkerque, France; 3 University Vasile Alecsandri, 600115 Bacau, Romania; 4 Organic Macromolecular Chemistry, Saarland University, Campus Saarbrücken C4 2, 66123 Saarbrücken, Germany

**Keywords:** cyclodextrins, inclusion compound, molecular modelling, space filling, static headspace gas chromatography, vapor pressure

## Abstract

The inclusion of volatile derivatives of benzene and cyclohexane in β-cyclodextrin (β-CD), hydroxypropyl-β-CD, and hydrophilic β-CD-thioethers was investigated by static headspace gas chromatography (HS-GC) and molecular modelling. The obtained binding constants strongly increase with the amount of space filling of the CD cavity and the salt concentration. β-CD thioethers show a 3–10 times higher binding potential than native β-CD.

## Introduction

β-Cyclodextrin (β-CD, **1**), the cyclic α(1→4) heptamer of glucose, is known to form inclusion compounds with a great variety of guests [1], such as derivatives of benzene [2–3], cyclohexane [4], adamantane [5–6], other alicyclic guests [7], and also inorganic molecules or ions [1,8].

Generally, the binding constant *K* increases with the degree of space filling of the β-CD cavity. The volume of the guest is often expressed by the number of carbon atoms *n* of its hydrophobic part. The Gibbs free enthalpy Δ*G°* of binding decreases nearly linearly with *n* by δΔ*G*^0^/δ*_n_* = −3.1 kJ mol^−1^ for linear alkanols [1]. Similar values were found for cyclic and polycyclic alkanoic acids δΔ*G*^0^/δ*_n_* = −3.3 kJ mol^−1^ [7]. We found an even higher slope for a series of *p*-substituted benzoic acids δΔ*G*^0^/δ*_n_* = −4.2 kJ mol^−1^, where the binding constant remarkably increased from *K* = 20 L mol^−1^ for benzoate to *K* = 18,400 L mol^−1^ for *p*-*tert*-butylbenzoate [3].

The observed increase of complex stability with increasing size of the hydrophobic part of the guest can be explained by the increase of hydrophobic interactions as well as other nonpolar interactions, e.g., van der Waals and dispersive interactions [9]. The molecular origin of hydrophobic interactions in general was already discussed controversially for many years [10–13]. Reliable quantitative predictive models are still missing [14]. Expulsion of “high energy water” from a cavity during space filling by a hydrophobic guest appears to be the main contributor to the driving force of complex formation [15]. The accumulation of basic understanding is essential for the formulation of precise docking programs, which estimate and screen the binding of drug candidates for biological receptors [16–17].

Practical applications of native β-CD, such as drug delivery [18–19], extraction of pollutants from soil [20–23], homogenous catalysis [24], and microbial degradation [25] are limited by both its low aqueous solubility (1.8 wt % at 25°C) and its moderate complexing ability. Especially hydrophobic guests form channel inclusion compounds with β-CD [26–27], which are nearly insoluble in water [28]. For instance the solubility of toluene in water is only 1.7 mM as a complex in β-CD compared to 5 mM in the free state.

Several β-CD derivatives have been synthesized to overcome these drawbacks. For instance, the statistical derivative hydroxypropyl-β-CD **2** was often employed, because it is much more water soluble than β-CD and can also be produced on an industrial scale [29–30]. Unfortunately, hydroxypropyl-β-CD **2** is not a single pure compound but a mixture of various similar homologues and isomers [31], and does not show formation constants superior to native β-CD. Thus, it was desirable to work with pure β-CD derivatives showing higher affinities.

**Figure 1:**
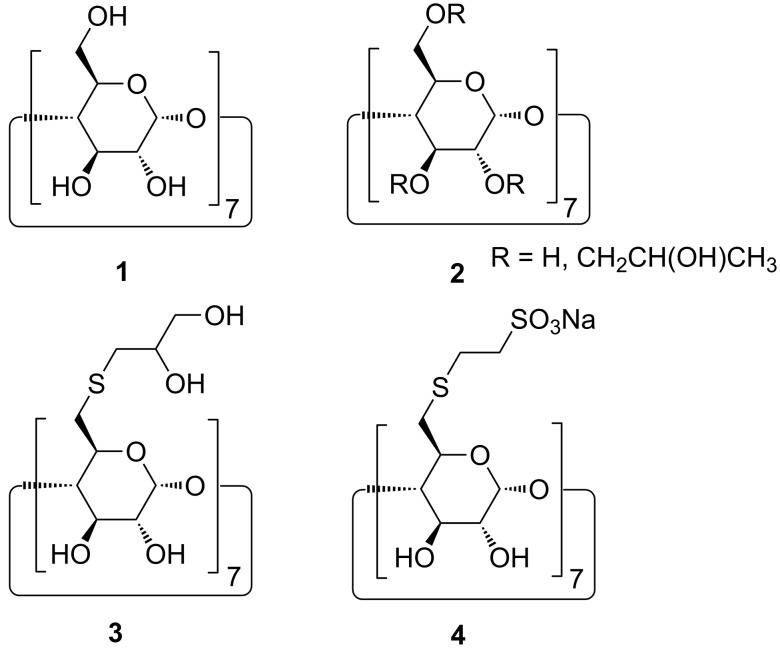
Chemical structures of β-CD **1**, hydroxypropyl-β-CD **2**, and β-CD-thioethers **3** and **4**.

We reported recently the synthesis and use of heptafunctional 6-S-substitued CD derivatives **3** and **4** (Figure 1) for the solubilization of sparingly water-soluble drugs [32–33]. These β-CD-thioethers are single pure compounds and they showed very promising binding constants and very high aqueous solubility.

In continuation of our investigations into the recognition of volatile organic compounds (VOCs) by CDs [34–35], we examined the potential of these β-CD-thioethers for the solubilization of VOCs. To demonstrate their versatility, two homologous series of volatile hydrophobic benzene and cyclohexane derivatives were chosen. These guests do not contain any hydrophilic substituents, therefore no attractive or repulsive polar interaction had to be taken into account.

Since static headspace gas chromatography (HS-GC) is a highly sensitive technique, it allows the investigation of the complexation of volatile guests in CD at very low concentrations, where guests, as well as their inclusion compounds, are still soluble in water [19,34–36]. Complexation of a guest is detected indirectly by the reduction of its vapor pressure due to complexation. This HS-GC method is applicable for a great variety of volatile guests [37].

The aim of this study was to investigate the binding ability of β-CD-thioethers toward hydrophobic VOCs in comparison to commercial β-CDs and to interpret the complexing abilities in terms of space filling of the cavities. Our systems are well-defined models for reaching a better understanding of hydrophobic interactions in general and are useful for the calibration of predictive tools.

## Results and Discussion

**Figure 2:**
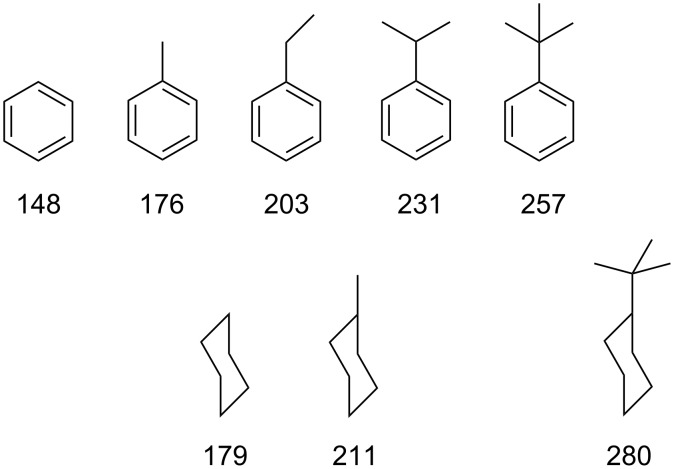
Chemical structures of the benzene and cyclohexane derivatives, with their calculated molecular volumes, *V*_G_ = *M*/*dN*_A_ in Å^3^, with *M* molecular weight, *d* density, *N_A_* Avogadro’s number.

A series of commercially available monosubstituted benzenes (Figure 2) was selected in analogy to the corresponding benzoates investigated previously [3].

**Figure 3:**
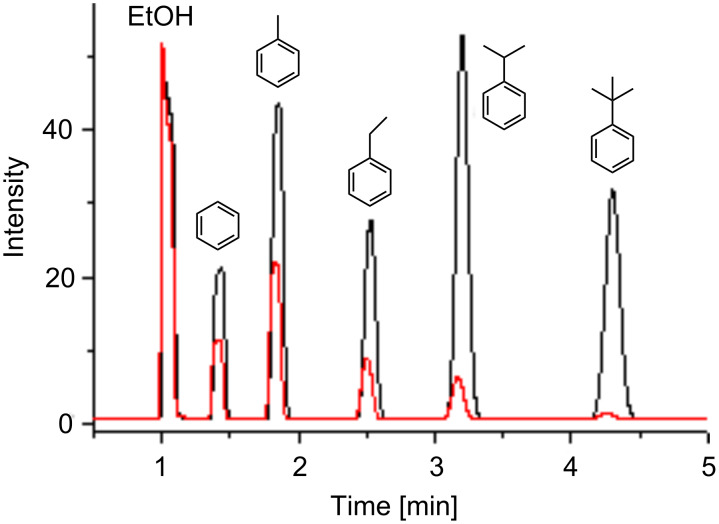
Chromatogram obtained by HS-GC for a mixture of aromatic guests (1 ppm) in water (black) and 10 mM solution of β-CD **1** (red).

These benzene derivatives were equilibrated in seated vials at very low concentrations (1 ppm) with solutions of β-CD derivatives (concentrations 1–10 mM). The partial pressures of these guests in the gas phase were determined by HS-GC. Because of the high separation efficiency of the GC column, all five guests could be determined simultaneously (Figure 3). Since interference of binding of the various guests was negligible because of the very large excess of β-CDs compared to the guests, binding constants could be determined for all of the five guests in parallel. The different decays of the signal intensities of the guests due to the complexation in β-CD are obvious in Figure 3.

The binding constant *K* was calculated from the ratio *y* = *A_0_*/*A* of the peak area of the guest without and with CD, *A_0_* and *A*, respectively, according to Equation 1, as derived in Supporting Information File 1 [36].

[1]
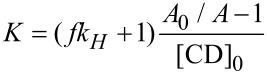


For a validation of the method, the whole concentration dependence of the integrated GC signal intensity was measured as a function of the CD concentration [CD]_0_ in the case of β-CD. The data points were fitted with the standard square root equation by nonlinear regression [19,34]. Within experimental error of ±10% the binding constant *K*, obtained by nonlinear regression, was identical to the value of *K* obtained according to Equation 1 from one data point. The latter method allows a fast and simultaneous determination of binding constant. The results for β-CD and the β-CD derivatives are summarized in Table 1 in comparison with values from the literature, which were in good agreement for β-CD **1** and in fair agreement for hydroxypropyl-β-CD **2** [2,4,29]. Deviations from literature values found for the statistical derivative **2** were attributed to slightly different substitution patterns.

**Table 1:** Binding constants *K* of benzene and cyclohexane derivatives in β-CD and β-CD derivatives as determined from HS-GC peak areas according to Equation 1.

guest\host	**1**	**2**	**3**	**4**	*n*^a^

	[M^−1^]	[M^−1^]	[M^−1^]	[M^−1^]	
benzene	128 (120^b^, 111^c^)	94 (99^c^)	942	226	6
toluene	158 (140^b^, 172^c^, 142^d^)	131 (170^c^, 163^d^)	1071	434	7
cyclohexane	341 (468^c^)	227 (363^c^)	4308	534	6
ethylbenzene	392 (330^b^, 289^c^)	303 (248^c^)	2285	1427	8
methylcyclohexane	295 (332^c^)	202 (253^c^)	4032	893	7
cumene	1684 (1200^b^)	1102	6992	6254	9
*tert*-butylbenzene	9503	1863	28081	24898	10
*tert*-butylcyclohexane	4092	2036	29345	44597	10

^a^*n* total number of carbon atoms; ^b^from [2], ^c^from [4], ^d^from [35].

The lower binding constant *K* of the host **2** compared to native β-CD was attributed to hydroxypropyl substituents at the 2- and 3-positions hindering the formation of intramolecular hydrogen bonds and thus destabilizing the β-CD framework. This destabilization leads to a reduction of the binding potential, because of an unfavorable negative binding entropy. Recently, we also found a diminished binding potential for those β-CD derivatives methylated at the 2- and 3-positions [38].

In contrast, the binding constants *K* of the β-CD thioethers were significantly higher by a factor of 3–10 than those of native β-CD. The higher binding potentials of the β-CD thioethers **3** and **4** compared to native β-CD could be attributed both to the higher hydrophobicity of sulfur compared to oxygen and to the exclusive localization of the substituents in position 6. This finding is consistent with previous observations of the high binding potentials of CD thioethers for other guests [32–33,39]. The binding constants of the neutral thioether **3** were in most cases higher than the ones of the anionic thioether **4**. The high hydrophilicity, introduced by the pendant sulfonate groups of **4**, seems to diminish the binding potential of the host.

**Figure 4:**
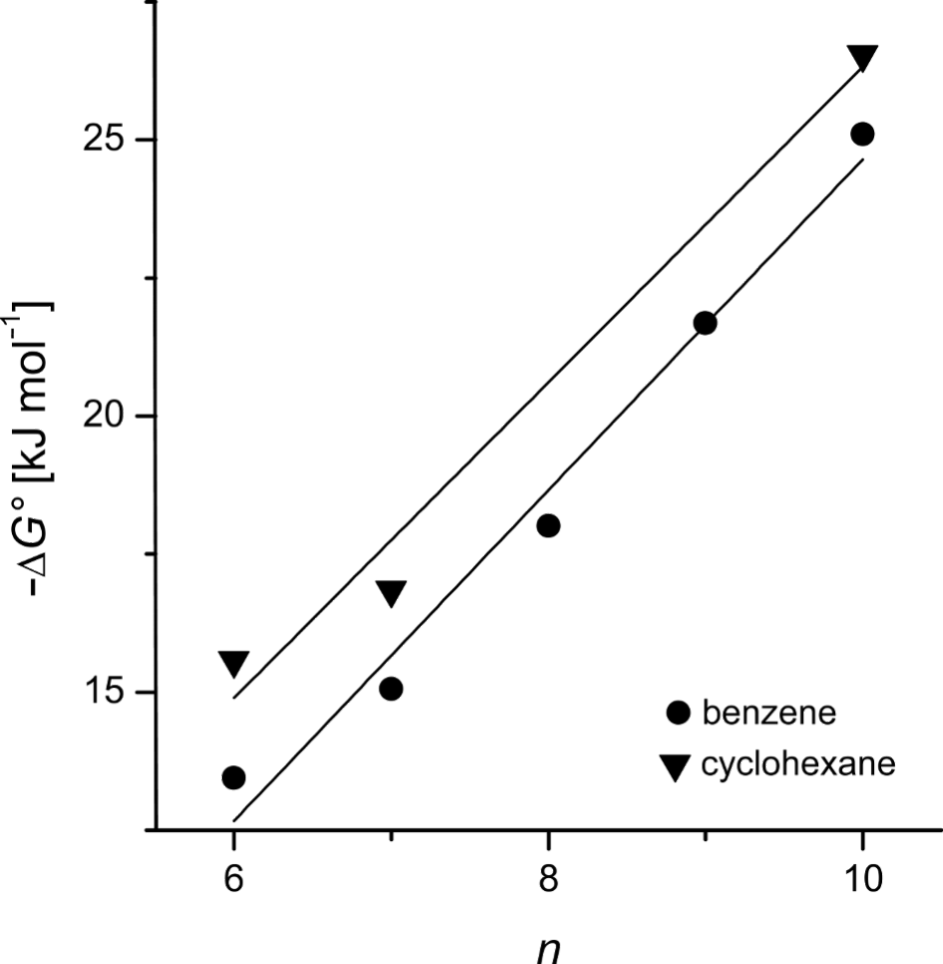
Gibbs free energy of formation of the inclusion compound of benzene derivatives, and cyclohexane derivatives in host **4** as a function of the total number of carbon atoms of the guest *n*.

Obviously, the binding constant *K* remarkably increased for all CD derivatives with the size of the guest, expressed by the number of carbon atoms *n*. The binding constant *K* = 128 M^−1^ for benzene in β-CD **1** was significantly higher than for benzoate *K* = 20 M^−1^ [3]. In contrast, the binding constant for *tert*-butylbenzene, *K* = 9,503 M^−1^ was lower than for *p*-*tert-*butylbenzoate, *K* = 18,400 M^−1^ [3]. The plot of Δ*G°* versus *n* was nearly linear (Figure 4). The slope δΔ*G*^0^/δ*_n_* = −2.9 kJ mol^−1^ was significantly smaller than the slope δΔ*G*^0^/δ*_n_* = −4.2 kJ mol^−1^ for the corresponding benzoate derivatives. This means that the carboxylate group at the guest can have either a repulsive or attractive interaction with β-CD, depending on the position of the carboxylate group within the β-CD cavity. The corresponding cyclohexane derivatives showed indeed a slope similar to the benzene derivatives, but free enthalpies more negative by ΔΔ*G°*= 2 kJ mol^−1^, which was attributed to the larger diameter of cyclohexane compared to benzene filling the β-CD cavity more completely.

**Figure 5:**
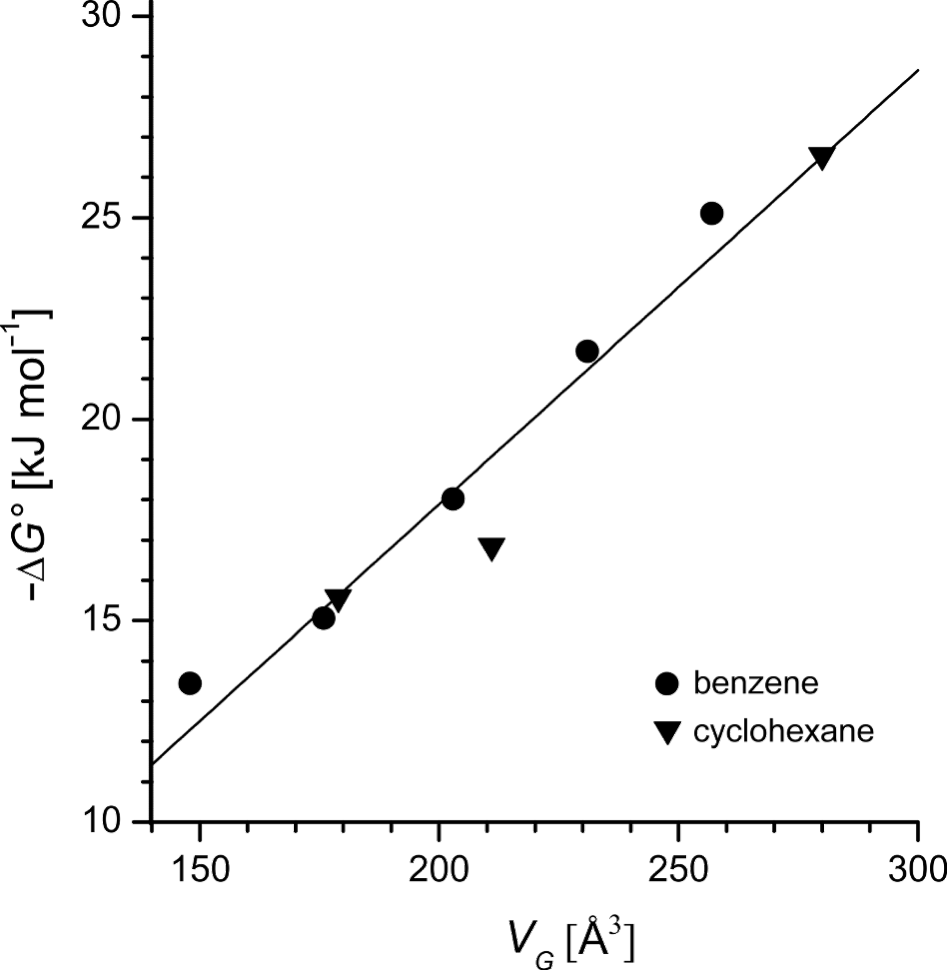
Gibbs free energy of formation of the inclusion compounds of benzene and cyclohexane derivatives in host **4** as a function of the molecular volume *V*_G_ = *M*/*dN*_A_.

For taking the space filling of the CD cavity better into account, the binding free energies Δ*G°* for host **4** were plotted as a function of the molecular volumes *V_G_* from Figure 2 of the guests (Figure 5). A reasonable linear master plot was obtained for all guests. The slope with the dimension of a pressure was *p* = −δΔ*G*^0^/δ*V*_G_ = 0.107 kJ mol^−1^Å^−3^ = 1.8 kbar. Hydrophobic interactions causing high internal pressures of this magnitude are already known to accelerate dimerization reactions, such as the Diels–Alder addition [40]. The molecular volume of the largest guest, *tert*-butylcyclohexane, *V*_G_ = 280 Å^3^ is close to the estimated volume of the β-CD cavity 262 Å^3^ [41], which means that the β-CD cavity within host **4**, extended by the thioether substituents, is nearly completely occupied by it.

**Figure 6:**
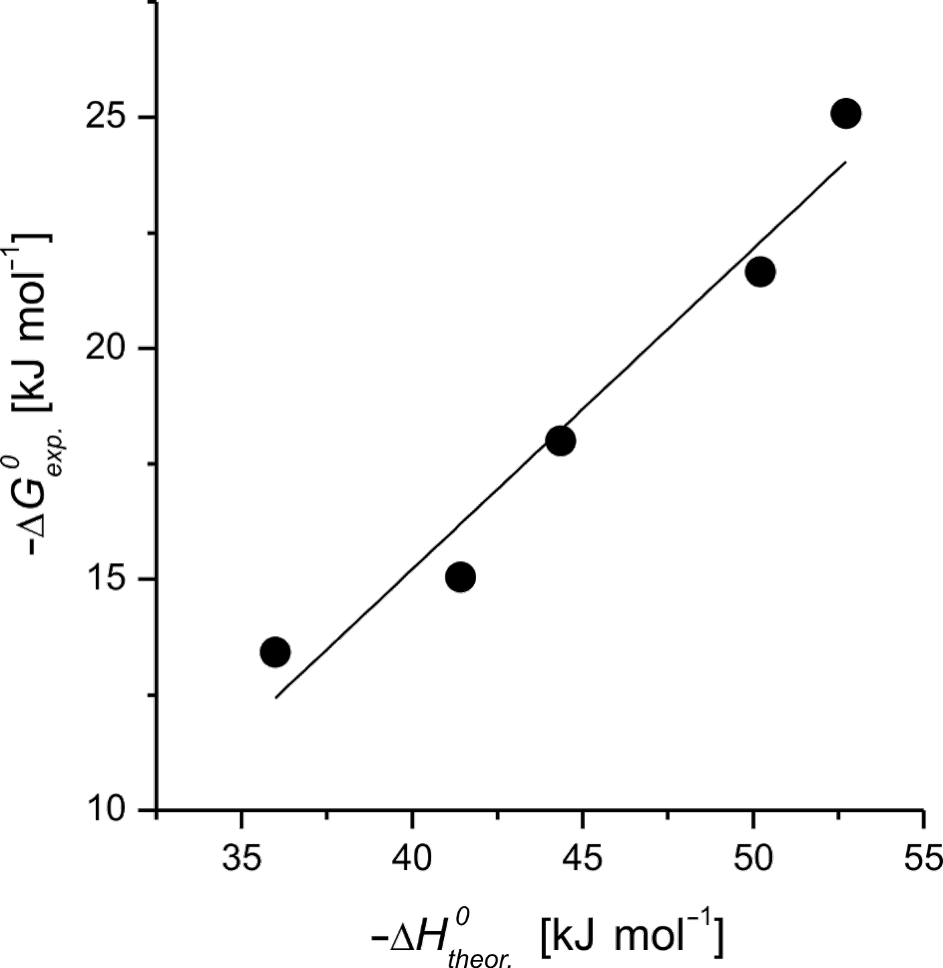
Gibbs free energy of formation of the inclusion compounds of benzene derivatives in host **4** as a function of the theoretical inclusion enthalpy, obtained by simulation by Macromodel (slope 0.69).

The ability of the program Macromodel (from Schrödinger Inc.) to predict the nonpolar interactions between host **4** and benzene derivatives was finally evaluated by means of docking simulations. Measured binding free energies were plotted as a function of the simulated inclusion enthalpies (Figure 6), defined as the enthalpy differences between the inclusion compounds and the free species. A significant linear correlation was obtained, confirming that the space filling of host cavity plays a dominant role within such series of complexes, and that the applied force field was reasonable. All guests fitted completely into the cavity of host **4** without steric hindrance. The slope of the experimental Gibbs free energy versus the simulated interaction enthalpy was still inferior to unity (0.69), which indicated that the host–guest interactions were partially compensated by reduced solvation and loss of freedom of the host–guest system.

**Figure 7:**
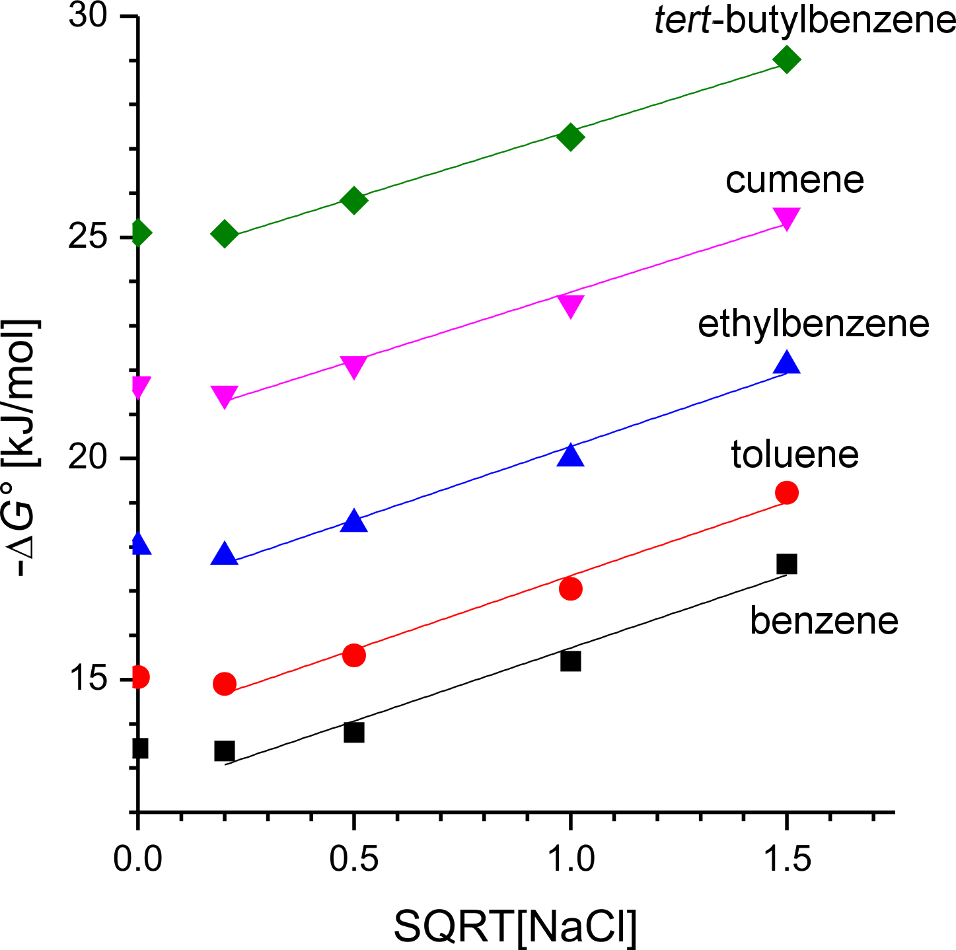
Free binding enthalpy for benzene, toluene, ethylbenzene, cumene, and *tert*-butylbenzene, in host **4** as a function of the square root of the salt concentration [NaCl].

One way to further improve the hydrophobic binding potential of a host in water is to add a salt such as NaCl [42]. From previous work we knew that –Δ*G°* increases linearly with the square root of the concentration of NaCl, equivalent to the ionic strength. We found a slope of


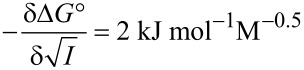


for binding in β-CD thioethers [43]. The investigation of the inclusion of the benzene derivatives in host **4** for various salt concentrations also provided a linear increase of the binding free enthalpy with the same slope as before,


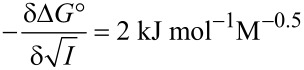


(Figure 7). Consequently, this salt effect appears to be a unique feature of hydrophobic binding, as neither the hosts nor the guests are expected to interact significantly with NaCl. Very high binding constants of up to 120,000 M^−1^ could be reached for *tert*-butylbenzene with host **4**.

## Conclusion

The GC-headspace technique is well suited for the determination of the stability constants of sparingly soluble inclusion compounds of volatile hydrophobic guests in CDs and their derivatives. β-CD thioethers **3** and **4** are good candidates for the solubilization of volatile hydrophobic compounds, because they are soluble in water at any concentration. Very high binding constants are obtained by attachment of hydrophobic substituents at both host and guest. However, substitution of CDs has to be performed at the primary positions of β-CD. Our binding data are well described by the space filling of the cavity, as defined either by the number of carbons *n*, the molecular volume of the guest *V_G_* or by molecular modeling.

## Experimental

### Materials

Benzene and cyclohexane derivatives were purchased from Aldrich, β-CD and hydroxypropyl-β-CD (DS 5.6) from Wacker Chemie. β-CD derivatives **3** and **4** were synthesized from β-CD **1** following published procedures. Distilled water was used in all experiments.

### Headspace analysis

Similar to those described in [44], measurements were conducted with an Agilent headspace autosampler. Sample solutions of 10 mL containing 1 ppm of guest were introduced into 20 mL headspace vials and sealed by using a silicone septa and aluminium foil. The vials were then thermostated at 25 ± 0.1 °C. After the equilibrium had been established (30 min), 1 mL of vapor from the above solution was withdrawn from the vial by using a gas-tight syringe and injected directly in the chromatographic column via a transfer line (250 °C). Each sample was then analyzed by gas chromatography (Perkin Elmer Autosystem XL equipped with a flame-ionization detector using a DB624 column). The GC settings were set as follows: detector temperature, 280 °C; column temperature, 120 °C during 5 min.

Full equilibrium of the chemical solute between liquid and gas phases is required. For the chemicals tested, the equilibrium time was 30 min. Linear response of the GC detector to the chemical concentration range tested is another requirement for the successful application of the method. For all compounds, we verify that the GC detector responds linearly. The correlation coefficients are all better than 0.998.

The determination of the Henry’s law constants were done with four different values of the aqueous volume *V* (3, 5, 7 and 10 mL for benzene derivatives and 7, 10, 12 and 15 mL for cyclohexane derivatives, respectively).

### Molecular modelling

Simulations of inclusion compounds for each benzene derivative into the cavity of host **4** were realized by means of Macromodel [45] with MMFF force field and GB/SA simulation of water [46]. The host structure was based on a nondistorted β-CD with C_7_ symmetry, on which primary hydroxyl groups were replaced by thioether arms (with a linear conformation, leading to a tubular extension of the cavity). Guests were constructed manually and submitted to minimization, prior to inclusion simulations. The docking of each guest inside host **4** was realized by means of Monte Carlo searches, with the generation of 5000 conformations (Polak–Ribiere conjugate gradient minimization, convergence fixed to 0.05 kJ Å^−1^ mol^−1^). During the search, host **4** was kept rigid, while the guest was freely modified. The most stable conformation for each inclusion compound was then completely relaxed (convergence fixed to 0.05 kJ Å^−1^ mol^−1^). Inclusion enthalpy was evaluated by the difference between the most stable inclusion-compound conformation and the free species.

## Supporting Information

File 1 Derivation of Equation 1 and determination of Henry’s law constant.
