# Supplementary material for: Space filling of β-cyclodextrin and β-cyclodextrin derivatives by volatile hydrophobic guests
Source: Beilstein J Org Chem. 2013 Jun 19;9:1185–91. doi: 10.3762/bjoc.9.133 (PMC3701372; doi:10.3762/bjoc.9.133)
Supplement: File 1 — Derivation of Equation 1 and determination of Henry’s law constant. [file Beilstein_J_Org_Chem-09-1185-s001.pdf]

Supporting Information

for

Space filling of β-cyclodextrin and β-cyclodextrin derivatives by volatile hydrophobic guests

Sophie Fourmentin[1,2], Anca Ciobanu[1,2,3], David Landy[1,2] and Gerhard Wenz*[,4]

Address: [1]University Lille Nord de France, F-59000 Lille, France, [2]ULCO, UCEIV, F-59140 Dunkerque, France, [3]University Vasile Alecsandri, 600115 Bacau, Romania and [4]Organic Macromolecular Chemistry, Saarland University, Campus Saarbrücken C4 2, 66123 Saarbrücken, Germany

Email: Gerhard Wenz* - g.wenz@mx.uni-saarland.de

*Corresponding author

Derivation of Equation 1 and determination of Henry's law constant

Derivation of Equation (1) [1]

Equation (1) was derived from the law of mass action (2), Henry's law (3), the mass balance, Equations (4, 5) and the Benesi–Hildebrand approximation (6) [2], which neglects the consumption of the host by the formation of the complex. This approximation is valid in our case because the total concentration of CD, $[CD]_0$, was several orders of magnitude higher than the concentration of the CD complex of the guest in the aqueous phase, [CD●G]. Henry's constant k_H, the ratio of the concentrations of the guest in the gas phase, $[G]^{gas}$, over the one in the aqueous phase, [G], was determined separately, as described in the section below. V and V^{gas} denote the volumes of the aqueous phase and the gas phase, respectively.

$$K = \frac{[CD \bullet G]}{[CD][G]} \cong \frac{[CD \bullet G]}{[CD]_0[G]} \quad (2)$$

$$k_H = \frac{[G]^{gas}}{[G]} \quad (3)$$

$$n_G^{total} = [G]^{gas} V^{gas} + [G]V + [CD \bullet G]V \quad (4)$$

$$c = n_G^{total}/V = f[G]^{gas} + [G] + [CD \bullet G] \quad \text{with} \quad f = V^{gas}/V \quad (5)$$

$$[CD] = [CD]_0 - [CD \bullet G] \cong [CD]_0 \quad (6)$$

$$c = fk_H[G] + [G] + K[CD]_0[G] = [G](1 + fk_H + K[CD]_0)$$

$$[G] = \frac{c}{1 + fk_H + K[CD]_0}$$

$$[G]^{gas} = \frac{k_H c}{1 + fk_H + K[CD]_0}$$

$$[G]_0^{gas} = \frac{k_H c}{1 + fk_H} \quad \text{for } [CD]_0 = 0$$

$$y = \frac{A_0}{A} = \frac{[G]_0^{gas}}{[G]^{gas}} = \frac{1 + fk_H + K[CD]_0}{1 + fk_H} = 1 + \frac{K}{1 + fk_H}[CD]_0$$

$$K = (fk_H + 1)\frac{y-1}{[CD]_0} = (fk_H + 1)\frac{A_0/A-1}{[CD]_0} \quad (1)$$

Determination of Henry's law constant k_H

We used the phase ratio variation (PRV) for the determination of Henry's law constant (k_H). This method is based on the following equation [3]:

$$\frac{C_0}{A} = \frac{1}{\alpha}\left(\frac{V^{gas}}{V} + \frac{1}{k_H}\right) \quad (7)$$

where C_0 is the initial chemical concentration in the prepared liquid solution (mg mL^{-1}), V is the aqueous sample volume added into the vial (mL), V^{gas} is the headspace gas volume in the vial (mL), A is the integrated area counts of GC peak for a given sample, and α is a specific parameter of the headspace, defined by $A = \alpha C_0$. In this study we used a fixed number of moles, n_0 so Equation (7) becomes:

$$\frac{1}{A} = \frac{1}{\alpha}\left(\frac{V^{gas}}{n_0} + \frac{V}{n_0 * k_H}\right)$$

$$\frac{1}{A} = m\frac{V}{V^{total}} + b$$

with $b = \frac{V^{total}}{\alpha * n_0}$ and $m = \frac{1}{\alpha * n_0}\left(-V^{total} + \frac{V^{total}}{k_H}\right)$

Linear regression of $1/A$ against V/V^{total} gives the slope and intercept. Henry's constant is calculated as $k_H = \frac{1}{m/b+1}$

Table 1: Henry's constant for benzene derivatives.

Guest\NaCl	0	0.04 M	0.25 M	1 M	2.25 M	4 M
benzene	0.27	0.27	0.29	0.43	0.68	0.85
toluene	0.30	0.30	0.35	0.52	0.91	1.07
ethylbenzene	0.36	0.36	0.44	0.67	1.26	1.35
cumene	0.50	0.50	0.64	0.97	1.97	1.91
tert-butylbenzene	0.55	0.55	0.70	1.08	2.26	2.20

Table 2: Henry's constant for cyclohexane derivatives.

Guest\NaCl	0	2.25 M
cyclohexane	7.05	7.35
methylcyclohexane	8.71	8.59
tert-butylcyclohexane	10.86	13.54

References

1. Lantz, A. W.; Wetterer, S. M.; Armstrong, D. W. *Anal. Bioanal. Chem.* **2005,** *383,* 160–166.
2. Benesi, H. A.; Hildebrand, J. H. *J. Am. Chem. Soc.* **1949,** *71,* 2703–2707.
3. Peng, J.; Wan, A. M. *Chemosphere* **1998,** *36,* 2731–2740.
